# High-throughput sequencing of insect specimens with sub-optimal DNA preservation using a practical, plate-based Illumina-compatible Tn5 transposase library preparation method

**DOI:** 10.1371/journal.pone.0300865

**Published:** 2024-03-22

**Authors:** Lauren Cobb, Erik de Muinck, Spyros Kollias, Morten Skage, Gregor D. Gilfillan, Markus A. K. Sydenham, Shuo-Wang Qiao, Bastiaan Star

**Affiliations:** 1 Centre for Ecological and Evolutionary Synthesis (CEES), Department of Biosciences, University of Oslo, Oslo, Norway; 2 Department of Medical Genetics, Oslo University Hospital Ullevål and University of Oslo, Oslo, Norway; 3 Norwegian Institute for Nature Research (NINA), Oslo, Norway; 4 Department of Immunology, Institute of Clinical Medicine, University of Oslo, Oslo, Norway; Bayero University Kano, NIGERIA

## Abstract

Entomological sampling and storage conditions often prioritise efficiency, practicality and conservation of morphological characteristics, and may therefore be suboptimal for DNA preservation. This practice can impact downstream molecular applications, such as the generation of high-throughput genomic libraries, which often requires substantial DNA input amounts. Here, we use a practical Tn5 transposase tagmentation-based library preparation method optimised for 96-well plates and low yield DNA extracts from insect legs that were stored under sub-optimal conditions for DNA preservation. The samples were kept in field vehicles for extended periods of time, before long-term storage in ethanol in the freezer, or dry at room temperature. By reducing DNA input to 6ng, more samples with sub-optimal DNA yields could be processed. We matched this low DNA input with a 6-fold dilution of a commercially available tagmentation enzyme, significantly reducing library preparation costs. Costs and workload were further suppressed by direct post-amplification pooling of individual libraries. We generated medium coverage (>3-fold) genomes for 88 out of 90 specimens, with an average of approximately 10-fold coverage. While samples stored in ethanol yielded significantly less DNA compared to those which were stored dry, these samples had superior sequencing statistics, with longer sequencing reads and higher rates of endogenous DNA. Furthermore, we find that the efficiency of tagmentation-based library preparation can be improved by a thorough post-amplification bead clean-up which selects against both short and large DNA fragments. By opening opportunities for the use of sub-optimally preserved, low yield DNA extracts, we broaden the scope of whole genome studies of insect specimens. We therefore expect these results and this protocol to be valuable for a range of applications in the field of entomology.

## Introduction

In response to widespread declines in insect diversity (reviewed in [1]), and the subsequent severe implications for ecosystem functioning (reviewed in [2]), large-scale international insect survey and sampling schemes have been put in place (e.g. [3]). While the primary interest of such schemes is to monitor abundance and distribution changes, the application of genomic approaches to insect specimens can also be used to investigate spatial drivers of population trends, habitat connectivity and local adaptation (reviewed in [4]). These approaches rely on high-throughput sequencing (HTS) methods, which generally have considerably greater DNA quality requirements compared to more traditional PCR-based methods. However, entomological samples are often collected and stored using a variety of techniques which form a trade-off between practicality, morphological requirements and molecular demands. While samples should ideally be stored immediately following tissue death under optimal conditions [5], this is not always feasible during extended field periods. For instance, entomological specimens are often temporarily stored in warm fieldwork vehicles until sampling has concluded, and those captured by high-efficiency trapping methods, such as pitfall and flight interception traps, can remain in traps on site for several days, or even weeks, prior to collection and processing [6]. In addition, as entomological specimens are important biological resources with myriad applications in the fields of taxonomy and ecology, standard collection and storage methods are designed to prioritise morphological preservation. Given these considerations, alcohols such as ethanol are commonly used as both killing agents and preserving fluids (e.g. [3]). While this approach helps to prevent physical deterioration, it does not fully inhibit DNA degradation due to relatively high water content [7, 8]. The use of highly concentrated ethanol (>96%) can improve DNA preservation, although ethanol concentration may decrease due to evaporative loss. In addition, entomological specimens preserved in high concentration ethanol can become overly brittle, impacting morphological analyses [9]. Ethanol-based sampling and storage nevertheless remains the cheapest and most practical method compared to other options such as flash freezing with liquid nitrogen, and is therefore widespread (e.g. [10–12]). Library preparation methods which are compatible with material collected and stored using suboptimal preservation protocols are therefore required when working with entomological specimens.

An additional obstacle to the use of entomological specimens for HTS is the destructive nature of DNA extraction: certain protocols require the sample be physically ground or beaten with beads, while most call for tissue digestion through the use of corrosive chemicals and enzymes (e.g. [13]). While it is possible to extract from the entire insect in order to prioritise DNA quantity and quality (see [14], this destructive approach requires the sacrifice of biologically valuable entomological specimens. Some ‘non-destructive’ insect DNA extraction methods have been proposed, whereby the entire specimen is incubated during the digestion step and subsequently retained, allowing for the preservation of the whole insect [15–18]. However, this practice may result in some morphological damage such as pigment loss [16, 19], and its impacts on long-term preservation (i.e. >10 years) remain unclear. Importantly, while re-extractions of the same biological material may produce viable genomic DNA extracts, the first extraction is often the most effective in terms of DNA yield and endogenous DNA content [20]. Using the entire specimen therefore limits any future DNA applications, reduces the long-term biological value of entomological collections, and fails to incorporate ethical guidelines for the destructive sampling of biological specimens [21]. To most effectively preserve insect specimens, for both future entomological applications and future DNA extractions, a more conservative solution would be to sacrifice a small piece of tissue such as a leg [20]. This approach leaves the majority of the insect untouched, although the low sample volume and consequent low DNA yield may constrain specific downstream molecular applications.

While the cost of whole genome sequencing has decreased by several orders of magnitude since its inception [22], library preparation often remains expensive and now represents a substantial proportion of the overall cost of whole genome data generation. For instance, methods whereby DNA is physically fragmented are widely adopted, yet can be expensive, time consuming, and often require high DNA input [23]. Alternatively, tagmentation-based library preparation has streamlined the process by combining DNA fragmentation and adapter ligation into a single reaction facilitated by Tn5 transposase, and typically calls for lower DNA input [24, 25]. Commercial Tn5 transposase kits, however, remain relatively expensive, and their dependence on the use of undisclosed reagents inhibits methodological optimisation. Several adaptations to the tagmentation method have been developed, such as the in-house production of Tn5 transposase [26–28]. Despite significantly reducing costs, in-house enzyme production can be both time and resource intensive, may not be always possible and therefore lacks broad-scale practicality. Further modifications include reduction of reaction volumes, replacement of reagents with cheaper alternatives and the incorporation of magnetic bead-linked transposomes, highlighting the potential of matching low DNA input amounts with diluted transposase concentrations to increase both throughput and affordability [25, 29, 30]. Here, we explore the use of a commercially available hyperactive Tn5 transposase (Diagenode) to create high-throughput sequencing libraries from low-yield DNA extracts obtained from entomological specimens.

Specifically, we investigate the effectiveness of a practical and in-house developed Tn5 tagmentation-based library preparation method, using insect DNA extracted by applying minimally destructive methods to samples initially stored in ethanol for several weeks while kept in fieldwork vehicles at ambient summer temperatures. After fieldwork was completed, single legs were removed from the specimens and subsequently stored in 96% ethanol at -18°C, while the remaining specimens were dried, pinned and stored at room temperature. Our study utilises samples stored using both methods. We assessed the robustness of the protocol in response to varying tagmentation reaction time and Tn5 transposase enzyme concentration. We then rescaled our protocol to fit 96-well plates and a standardised DNA input of 6ng. Finally, we developed a double-sided post-amplification clean-up protocol in order to optimise library fragment size distribution and thus improve sequencing efficiency. We find that this economical, easily scalable library preparation protocol opens multiple opportunities for genomic studies, particularly in the cases of non-model organisms with limited funding, small organisms with low per-sample DNA yield or biological samples stored under non-optimal conditions for DNA preservation.

## Materials and methods

### Specimens, sampling and storage conditions

122 samples of two different bumblebee species (*Bombus lapidarius* and *Bombus pascuorum*) were stored following one of two protocols (see Table 1). All specimens were sampled in 2017 and 2022 by immediate submersion in 96% ethanol. They were then stored and transported in fieldwork vehicles for several weeks during the summer, before being dried, pinned and stored at room temperature with no preservative (Dry Protocol, *n* = 82). Samples stored using the Ethanol Protocol (*n* = 40) were comprised of single legs which were removed from the specimens collected in 2017 prior to drying, and subsequently stored in 96% ethanol at -18°C.

**Table 1:** Bumblebee specimen sampling and storage features. Details of two bumblebee sample storage protocols, including year sampled, killing agent, storage preservative and storage temperature, and the number of legs used for DNA extraction. Also presented are the sample sizes from each group used in two different sets of analyses: DNA concentration comparison of DNA extracts, and library preparation and sequencing statistics.

Protocol	Year sampled	Killing agent	Storage preservative	Temperature	Specimens (*n*)	One leg extracted (*n*)	Two legs extracted (*n*)	DNA concentration comparison (*n*)	Library preparation and sequencing (*n*)
Dry	2017 / 2022	Ethanol	None	Ambient	82	20	62	20	82
Ethanol	2017	Ethanol	Ethanol	-18°C	40	40	0	40	8

### DNA extraction

DNA was extracted from either 1 or 2 mid or hind legs, including the coxa, femur, tibia, and tarsomeres, using the Qiagen DNeasy Blood & Tissue Kit. Samples from the Ethanol Protocol were washed with molecular-grade water in order to remove any ethanol residue. All samples were incubated in 380μL digestion buffer (300μL ATL buffer, 50μL DTT, 30μL proteinase K) at 56° C and 350 RPM overnight (minimum 17 hours), as implemented by the Campos & Gilbert method (in [31]) for DNA extraction from chitin. Following overnight digestion, 7μL RNase A and 300μL AL buffer were added to the lysate, followed by a room temperature incubation (15 minutes) and the addition of 360 μL 96% ethanol. DNA was subsequently bound and purified following the manufacturer’s recommendations, then eluted in AE buffer (105μL, 90μL or 80μL) and stored at -18°C. DNA concentration was then estimated using the Qubit dsDNA Assays (Thermofisher) and standardised to 1ng/μL.

Initially, DNA extractions of dry-stored specimens were performed on single legs (*n* = 21). In order to consistently avoid very low DNA yields, two legs were used for the remaining 61 extractions, along with the addition of 5μL 1M CaCl_2_ solution to the digestion buffer to aid tissue digestion.

### Library preparation

Two 8-sample Illumina compatible trial libraries were generated to evaluate the impact of two variables on DNA fragment size distribution: 1) tagmentation reaction time (Trial 1) and 2) Tn5 transposase enzyme concentration (Trial 2). For Trial 1, two *B*. *pascuorum* DNA extracts were incubated for five, six, seven or eight minutes with 1μL of undiluted Loaded Tn5 Tagmentase (Diagenode, C01070012). For trial two, the same DNA extracts were incubated with diluted Loaded Tn5 Tagmentase (either a 2X, 4X, 6X or 8X-fold dilution of the original Tn5 Tagmentase) for 7 minutes.

Following these trials, the protocol was adapted for a 96-well plate setup, and 96 libraries were generated using a 7 minute incubation and 6X Loaded Tn5 Tagmentase dilution. The libraries were built using extracts with DNA concentrations of at least 1ng/μL, consisting of 82 dry-stored samples, 8 ethanol-stored samples, and 6 additional samples which were either found to be misidentified bumblebee species, or stored using a different protocol. Following PCR amplification, the libraries were pooled and cleaned using two alternative clean-up protocols (Pool SP and Pool S4) in order to optimise fragment size distribution for sequencing (see also S1 Protocol for full Tn5 tagmentation library preparation, PCR and post-amplification clean-up protocol.).

### Tn5 tagmentation library preparation and PCR

All DNA extracts were standardised to 1ng/μL. 6μL DNA extract were incubated (55°C) with 5μL of 2X Tagmentation Buffer (Diagenode, C01019043) and 1μL of Loaded Tn5 Tagmentase (Diagenode, C01070012, using various dilutions, see above). After 5, 6, 7 or 8 minutes (see above), 3μL 0.2% Sodium dodecyl sulfate (SDS) Solution (Molecular Biology/Electrophoresis, Fisher BioReagents™ MFCD00036175) was added to stop the tagmentation reaction, followed by a 5 minute room temperature incubation. Post-tagmentation PCR (12 cycles) was done in 40μL reactions using KAPA HiFi DNA Polymerase (HotStart and Ready Mix formulation, Roche, KK2601). The manufacturer’s recommendations were followed by adding 24μL Mastermix, 1μL N7 Primer (1μM) and 1μL N5 Primer (1μM) (IDT for Illumina UD Indexes Plate A/Set 1, Illumina) to each 15μL reaction. After amplification, 6μL of each of the 96 wells was pooled (without quantification) before proceeding to clean-up (AMPure XP, Beckman Coulter).

### Post-amplification clean-up and sequencing

All bead clean-ups were performed using AMPure XP beads (Beckman Coulter). The Trial 1, Trial 2 and Pool SP libraries were cleaned once, removing small DNA fragments by using a 0.6 DNA/bead ratio, following the manufacturer’s recommendations. Pool S4 was built from the same PCR reaction as Pool SP, but cleaned twice, using different bead ratios to remove both large and small DNA fragments (as demonstrated in [32]), with a target median fragment size distribution of ~600bp. Firstly, a 0.5 DNA bead/ratio was used to remove large DNA fragments. Secondly, a 0.65 DNA/bead ratio was used to remove smaller DNA fragments and shift the fragment size distribution to peak between 500 and 1000 bp. This “double sided” clean-up process was performed twice, and the final library was eluted in 40μL molecular grade water. DNA fragment length for all amplified libraries (individual libraries for Trial 1 and Trial 2, Pool SP, and Pool S4) were analysed using a Fragment Analyzer™ (Advanced Analytical) with the DNF-474 High Sensitivity Fragment Analysis Kit after 10-fold dilution of library aliquots. Pool SP was sequenced using an Illumina Novaseq 6000 SP flowcell, generating 923,253,536 read pairs passing filters and Pool S4 was sequenced using an Illumina Novaseq 6000 S4 flowcell and generated 2,799,713,996 read pairs passing filters. Base calling and demultiplexing were performed with RTA v3.4.4 and bcl2fastq v2.20.0.422 in both cases.

Read data were aligned using PALEOMIX v1.2.14 [33] to BomLapEIv1 (https://www.ncbi.nlm.nih.gov/datasets/genome/GCA_936014575.1/) and BomPasc1.1 [34] after using AdapterRemoval/2.3.1 [35] and the mem algorithm as implemented in BWA/0.7.17 [36]. Only reads with a minimum MapQ value of 15 were retained and considered endogenous.

### Statistics

The DNA yield comparison between dry-stored and ethanol-stored specimens was carried out using a t-test. Mann-Whitney-Wilcoxon tests were used to compare sequencing statistics between a) dry-stored and ethanol-stored specimens and b) Pool SP and Pool S4, excluding the endogenous DNA comparison between Pool SP and Pool S4, for which a t-test was used. All statistical analyses were performed using base R 4.3.1.

### Ethics statement

There are no legal limitations on sampling *B*. *pascuorum* and *B*. *lapidarius* in Scandinavia. Bees in Germany were collected under permit LLUR 515 from the Schleswig-Holstein Nature Conservation Bureau, issued to M. Kuhlmann.

## Results

### Sample storage impact on DNA yield

**Fig 1:**
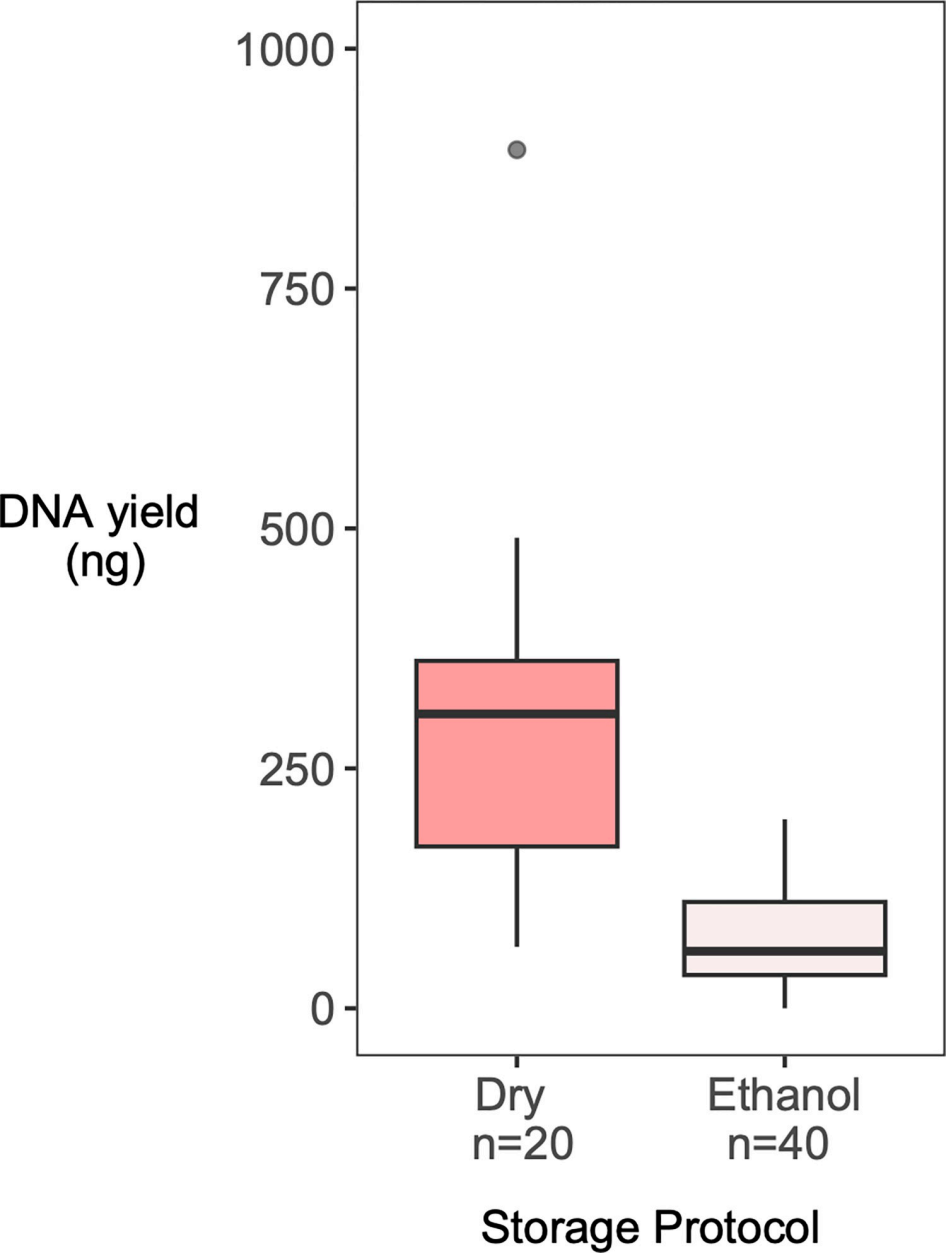
DNA extract concentration measurements (ng) from single bumblebee legs (*B*. *pascuorum* and *B*. *lapidarius*) stored using 2 different methods. Specimens were sampled in 2017 from various locations in Norway, Germany and Denmark and stored in ethanol in fieldwork vehicles at ambient summer temperatures for several weeks. Samples were then either stored dry (at room temperature) or in ethanol (at -18°C). See Materials and Methods for details regarding sample collection and DNA extraction. Following extraction, DNA was eluted in 105μL. DNA yields from dry-stored specimens (mean = 298.21±181.03) were significantly greater than from those stored in ethanol (mean *=* 72.98±51.79; t = 5.45, df = 20.57, P < 0.001).

One leg was used to extract DNA from samples stored in ethanol at -18°C (*n* = 40) and either one leg (*n* = 20) or two legs (*n* = 62) were used from samples stored dried at ambient temperatures (Table 1). When comparing DNA yields of extracts generated from comparable tissue volumes (a single leg), we found that significantly more DNA is obtained from the dry-stored specimens than the ethanol-stored specimens (Fig 1). Given that DNA yields from ethanol-stored specimens were low, we chose to prioritise dry-stored specimens for library preparation.

### Tn5 transposase library preparation optimisation

**Fig 2:**
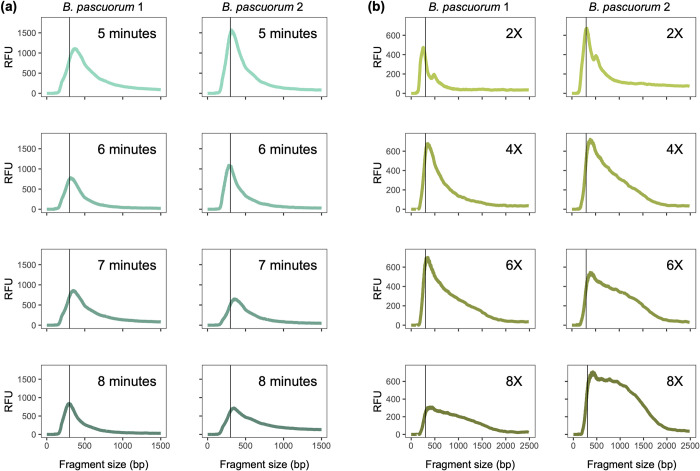
Fragment size distribution profiles of DNA libraries built from a standardised input of 6ng DNA from each of two *B*. *pascuorum* specimens, using different tagmentation reaction times and transposase dilutions. **(a)** Libraries were built using four different tagmentation reaction times (5 minutes; 6 minutes; 7 minutes; 8 minutes) with a 1X transposase dilution. **(b)** Libraries were built using four different transposase dilutions (2X; 4X; 6X; 8X) with a 7 minute tagmentation reaction. RFU (Relative Fluorescence Units) signifies relative amount of DNA. A vertical line indicating a fragment size of 300 bp is included to assist visual comparison of the different treatments. See Materials and Methods for details regarding library preparation protocol.

Changing tagmentation reaction time had minimal impact on fragment size distribution, with all libraries showing similar distribution profiles and fragment size peaking slightly above 300 bp (see Fig 2A). Fragmentation size distribution profiles of libraries built using 2X, 4X and 6X transposase enzyme dilutions were broader, and showed a modest increase in the proportion of long DNA fragments with each ascending dilution. This increase becomes more pronounced with the use of an 8X dilution (see Fig 2B). We therefore generated libraries in a 96-well plate using a 7 minute incubation time and 6X Loaded Tn5 Tagmentase dilution. These libraries were then amplified (12 cycles), after which 5μL from each library was pooled to perform bead clean-up on all libraries combined using two different approaches.

### Post-amplification clean-up optimisation

**Fig 3:**
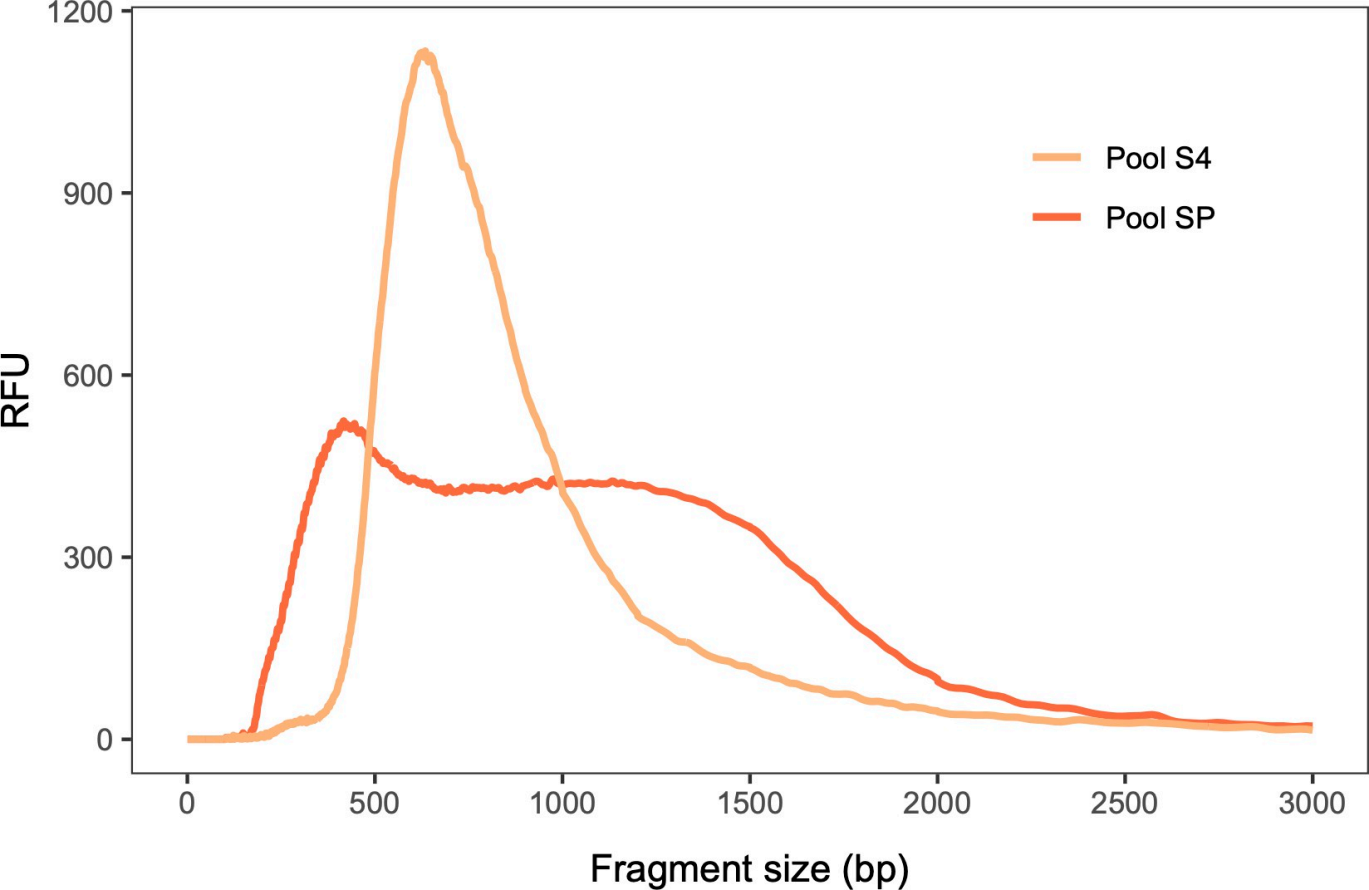
Comparison of fragment size distribution of 96 pooled bumblebee DNA libraries which were cleaned up with different AMPure XP protocols: A single clean-up with a 0.6 DNA/bead ratio (Pool SP), and a double-sided clean-up using two ratios (0.5 DNA/bead ratio followed by 0.65 DNA/bead ratio) which was performed twice (Pool S4). RFU (Relative Fluorescence Units) signifies relative amount of DNA.

Following initial bead clean-up, we obtained a fragment size distribution situated between 400 bp and 1500 bp (Pool SP, Fig 3), a much broader range than the tight distribution around 600 bp recommended for Illumina DNA sequencing [37]. Pool SP was sequenced on an Illumina Novaseq SP flowcell with paired-end 150 bp reads, with a large number of reads collapsing into a single read (see further below). We therefore performed a more stringent clean-up on the original pooled libraries to tighten fragment size distribution, removing both very short and very long DNA fragments, resulting in a narrow distribution peaking around 600 bp (Pool S4, Fig 3). Pool S4 was subsequently sequenced on one quarter of an Illumina 6000 S4 flowcell, also with paired-end 150 bp reads.

### Sequencing results

**Fig 4:**
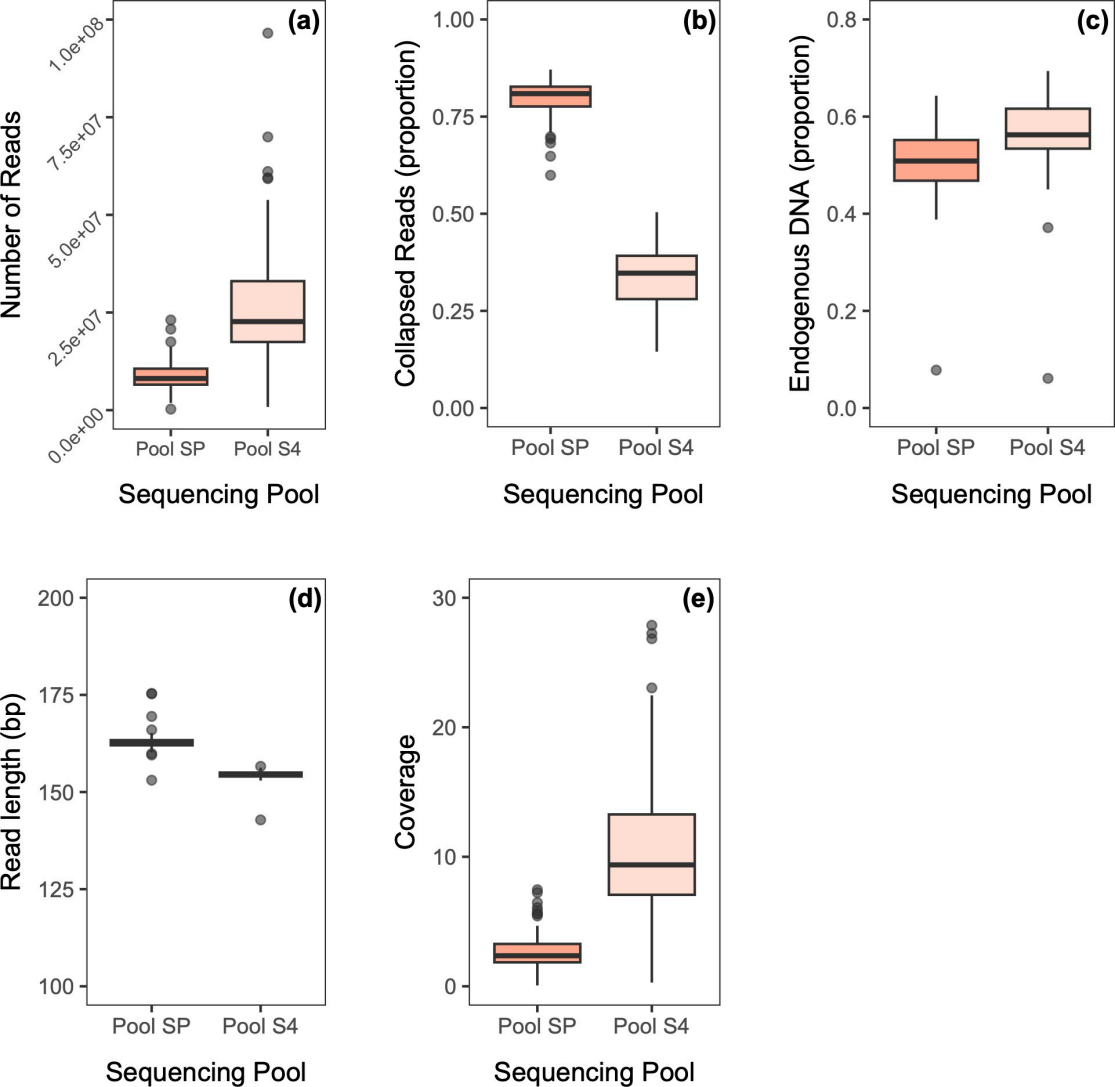
Sequencing comparisons of two pools of 90 bumblebee libraries. Bumblebee specimens were sampled between 2017 and 2022 from various locations in Norway, Germany, Denmark and Sweden. Both pools were generated from the same parallel library session. Pool SP was cleaned up with a 0.6 DNA/bead ratio and was sequenced on an Illumina Novaseq SP flowcell. Pool S4 was cleaned up using a double-sided size selection protocol, with a 0.5 DNA/bead ratio clean-up followed by a 0.65 DNA/bead ratio clean-up (twice) and was sequenced on ¼ Illumina Novaseq S4 flowcell. The boxplots compare **(a)** number of reads; **(b)** proportion of collapsed reads; **(c)** endogenous DNA proportion (unique reads only); **(d)** read length and **(e)** coverage (fold).

We obtained 795,694,049 reads ranging between 261,576 and 23,087,451 per specimen for Pool SP, while Pool S4 produced significantly more reads: 2,409,119,984, ranging between 835,001 and 96,527,020 per specimen (W = 429, P = <0.001; Table 2 and Fig 4A). Furthermore, Pool SP contained a significantly higher number of collapsed reads (W = 8100, P = <0.001; Fig 4B), slightly lower endogenous DNA proportion (t = -25.89, df = 89, P = <0.001; Fig 4C), and slightly higher overall read length (W = 8012, P = <0.001; Fig 4D) compared to Pool S4. Overall, Pool S4 resulted in higher coverage than Pool SP (W = 365, P = <0.001; Fig 4E), which was predominantly driven by the higher sequencing yield (Fig 4A). Nonetheless, when calculating the ratio of coverage against sequencing yield (calculated as coverage divided by number of paired reads), the coverage achieved by Pool S4 was on average greater than that of Pool SP by a factor of 1.45±0.12. This increase in efficiency for Pool S4 can be explained by both its reduced proportion of collapsed reads, leading to the mapping of more nucleotides per pair, and its higher proportion of endogenous DNA, leading to more unique mapped sequences per pair.

**Table 2:** a & b. Sequencing statistics for two sequencing pools (SP and S4) of 90 specimens of two bumblebee species: (a) *Bombus lapidarius* (*n* = 46) and (b) *Bombus pascuorum* (*n* = 44). Bumblebee specimens were sampled between 2017 and 2022 from various locations in Norway, Germany, Denmark and Sweden. Libraries for both pools were generated simultaneously. The SP pool was cleaned up with a 0.6 DNA/bead ratio and was sequenced on an Illumina Novaseq SP flowcell. The S4 pool was cleaned up using a double-sided size selection protocol, with a 0.5 DNA/bead ratio clean-up followed by a 0.65 DNA/bead ratio clean-up (twice) and was sequenced on ¼ Illumina Novaseq S4 flowcell. Statistics presented for each sequencing pool are specimen ID, number of reads, collapsed reads (proportion), clonality (proportion), endogenous DNA (proportion), nuclear coverage (fold), and read length (bp). Also presented is combined per-specimen coverage of both sequencing runs.

**(a)**													
***Bombus lapidarius***													
**ID**	**SP**						**S4**						**Combined** **coverage (fold)**
	**Reads**	**Collapsed reads** **(proportion)**	**Clonality** **(proportion)**	**Endogenous DNA** **(proportion)**	**Coverage** **(fold)**	**Read length** **(bp)**	**Reads**	**Collapsed reads** **(proportion)**	**Clonality (proportion)**	**Endogenous DNA** **(proportion)**	**Coverage** **(fold)**	**Read length** **(bp)**	
BEE009	9974301	0,871	0,066	0,470	2,397	160,435	19387911	0,329	0,117	0,536	7,659	156,283	10,056
BEE010	9973152	0,842	0,071	0,470	2,458	160,446	25403974	0,377	0,131	0,514	9,263	154,988	11,721
BEE014	8076852	0,831	0,067	0,481	2,092	163,328	20241708	0,326	0,121	0,550	8,167	155,345	10,259
BEE016	12203996	0,829	0,071	0,479	3,146	162,998	28735826	0,328	0,124	0,545	11,470	155,234	14,616
BEE017	9640662	0,838	0,072	0,460	2,362	162,387	22589207	0,356	0,129	0,509	8,263	155,072	10,625
BEE018	7609357	0,860	0,067	0,478	1,889	161,304	16140277	0,361	0,117	0,550	6,398	155,828	8,287
BEE019	12168745	0,803	0,067	0,452	3,039	163,656	36439272	0,368	0,124	0,506	13,112	154,294	16,151
BEE021	16261719	0,726	0,054	0,588	5,604	162,840	53827117	0,205	0,113	0,641	26,837	153,707	32,441
BEE025	23087451	0,740	0,063	0,556	7,459	163,406	96527020	0,241	0,147	0,612	45,061	153,617	52,520
BEE027	14102012	0,796	0,066	0,587	4,514	160,541	34124445	0,189	0,120	0,639	17,289	155,188	21,803
BEE032	13389169	0,847	0,071	0,465	3,276	161,618	23670306	0,314	0,120	0,534	9,339	155,415	12,615
BEE034	20745911	0,749	0,076	0,539	6,463	163,746	69948180	0,215	0,169	0,589	31,912	153,800	38,375
BEE037	12311833	0,785	0,055	0,566	3,878	162,403	31316323	0,203	0,106	0,620	15,312	155,496	19,190
BEE038	6629450	0,828	0,066	0,436	1,564	163,612	20515904	0,459	0,122	0,473	6,519	154,457	8,083
BEE039	9868829	0,811	0,077	0,428	2,317	163,437	27932752	0,394	0,133	0,482	9,416	154,365	11,732
BEE040	13433561	0,818	0,072	0,506	3,686	162,638	28159433	0,276	0,123	0,585	12,419	154,880	16,105
BEE041	10106362	0,813	0,083	0,448	2,489	164,142	43363140	0,435	0,161	0,489	14,436	154,196	16,924
BEE043	5924824	0,833	0,068	0,461	1,446	160,913	16846127	0,455	0,121	0,493	5,605	154,640	7,051
BEE047	14470649	0,718	0,070	0,643	5,430	161,472	42097600	0,167	0,123	0,670	22,463	153,943	27,893
BEE165	11932018	0,742	0,067	0,527	3,655	163,741	47903029	0,280	0,144	0,562	20,086	153,714	23,741
BEE166	10186140	0,774	0,061	0,541	3,070	161,137	26039416	0,268	0,097	0,616	12,096	154,291	15,166
BEE168	10210866	0,736	0,057	0,442	2,619	162,861	36326853	0,222	0,108	0,473	13,270	153,865	15,889
BEE178	13903566	0,741	0,060	0,492	3,941	162,279	59516346	0,388	0,126	0,541	22,442	153,188	26,383
BEE254	8588713	0,821	0,065	0,462	2,139	162,012	24208707	0,373	0,112	0,522	8,975	154,743	11,114
BEE255	8851361	0,820	0,063	0,474	2,280	163,271	25350112	0,361	0,113	0,538	9,781	155,012	12,062
BEE256	7091677	0,820	0,068	0,454	1,753	163,622	22236744	0,435	0,123	0,500	7,595	154,543	9,348
BEE257	2427508	0,770	0,084	0,497	0,641	153,048	8916584	0,391	0,111	0,569	3,292	142,826	3,933
BEE258	8132019	0,824	0,061	0,477	2,097	162,749	30014805	0,302	0,114	0,538	12,016	155,311	14,113
BEE259	7600369	0,814	0,080	0,455	1,885	162,846	22660080	0,396	0,145	0,498	7,892	154,618	9,778
BEE260	7354599	0,682	0,081	0,078	0,341	159,535	25988804	0,145	0,140	0,061	1,280	153,194	1,621
BEE261	14375044	0,818	0,074	0,474	3,715	163,360	37035119	0,352	0,131	0,547	14,558	154,671	18,273
BEE263	10276519	0,803	0,059	0,462	2,616	162,954	28463838	0,369	0,103	0,534	10,798	154,208	13,414
BEE264	7120018	0,831	0,066	0,462	1,768	163,087	18442004	0,436	0,111	0,509	6,408	154,634	8,176
BEE265	5453553	0,823	0,062	0,468	1,384	163,422	16730402	0,460	0,109	0,511	5,740	154,453	7,124
BEE266	6616852	0,809	0,070	0,420	1,525	163,393	17800519	0,409	0,120	0,450	5,556	154,502	7,081
BEE267	5917461	0,819	0,058	0,468	1,499	162,297	17694985	0,344	0,092	0,551	7,066	155,077	8,565
BEE268	6664674	0,807	0,056	0,501	1,838	163,453	19275461	0,381	0,104	0,550	7,491	154,678	9,330
BEE269	7465422	0,810	0,066	0,470	1,921	163,122	17086497	0,392	0,106	0,527	6,306	154,312	8,227
BEE270	261576	0,798	0,056	0,455	0,067	165,995	835001	0,440	0,078	0,505	0,288	155,124	0,355
BEE271	9085590	0,836	0,072	0,463	2,243	162,363	19003806	0,358	0,118	0,537	7,332	155,177	9,575
BEE272	2456658	0,804	0,057	0,466	0,634	164,139	7233437	0,452	0,090	0,511	2,495	154,413	3,129
BEE273	5807791	0,798	0,057	0,430	1,385	163,551	20537471	0,504	0,109	0,463	6,164	153,630	7,548
BEE274	6422043	0,831	0,055	0,489	1,682	162,351	13546114	0,323	0,092	0,564	5,616	155,304	7,298
BEE275	11070844	0,820	0,066	0,468	2,813	163,006	5837269	0,402	0,086	0,544	2,212	154,424	5,025
BEE276	7392200	0,809	0,063	0,480	1,944	163,083	22690095	0,358	0,117	0,541	8,786	154,502	10,730
BEE277	5956503	0,836	0,057	0,447	1,422	162,493	17784587	0,463	0,097	0,502	5,984	154,540	7,406
**(b)**													
***Bombus pascuorum***													
**ID**	**SP**						**S4**						**Combined coverage (fold)**
	**Reads**	**Collapsed reads (proportion)**	**Clonality (proportion)**	**Endogenous DNA (proportion)**	**Coverage (fold)**	**Read length (bp)**	**Reads**	**Collapsed reads (proportion)**	**Clonality (proportion)**	**Endogenous DNA (proportion)**	**Coverage (fold)**	**Read length (bp)**	
BEE008	6549854	0,862	0,056	0,574	2,230	160,364	14692031	0,289	0,108	0,657	8,376	156,090	10,605
BEE011	9454360	0,811	0,066	0,547	3,251	162,735	28641027	0,338	0,116	0,625	14,955	154,549	18,207
BEE012	1842509	0,648	0,039	0,560	0,796	175,369	12755700	0,316	0,106	0,537	5,768	153,834	6,564
BEE013	9979839	0,855	0,072	0,604	3,597	160,225	19615704	0,256	0,116	0,694	12,048	156,115	15,645
BEE015	6242105	0,847	0,056	0,620	2,348	161,792	15278897	0,255	0,109	0,693	9,411	156,607	11,759
BEE020	17478850	0,731	0,064	0,620	7,235	161,673	59277548	0,195	0,127	0,665	35,583	153,820	42,818
BEE022	13710915	0,698	0,056	0,595	5,601	162,110	61087559	0,185	0,126	0,634	35,015	153,281	40,615
BEE023	6881537	0,796	0,080	0,532	2,338	163,275	20380076	0,338	0,130	0,598	10,186	154,551	12,524
BEE024	6102715	0,774	0,050	0,600	2,476	169,461	18644941	0,230	0,101	0,670	11,108	154,536	13,583
BEE028	6652996	0,862	0,071	0,528	2,076	159,861	14249671	0,359	0,116	0,604	7,161	155,907	9,238
BEE031	10012093	0,793	0,063	0,508	3,255	162,919	29458652	0,294	0,120	0,567	14,298	154,369	17,553
BEE033	6172151	0,850	0,063	0,560	2,084	161,214	13102527	0,304	0,103	0,650	7,324	155,882	9,408
BEE035	4694766	0,809	0,066	0,504	1,504	164,292	17189720	0,422	0,127	0,542	7,365	154,092	8,870
BEE036	7118608	0,796	0,064	0,502	2,285	163,293	26096664	0,347	0,117	0,565	12,219	154,174	14,504
BEE042	7127099	0,794	0,062	0,527	2,391	162,470	24946535	0,388	0,111	0,591	11,895	154,038	14,286
BEE044	10297225	0,802	0,062	0,574	3,711	161,244	24511141	0,287	0,103	0,653	13,801	154,889	17,512
BEE045	6629392	0,842	0,055	0,534	2,169	162,890	18638735	0,348	0,111	0,603	9,375	155,248	11,544
BEE046	9300192	0,771	0,066	0,515	3,112	162,764	36373177	0,412	0,121	0,563	16,234	153,618	19,346
BEE048	11154948	0,782	0,059	0,553	3,962	162,237	34077198	0,282	0,107	0,628	18,432	154,256	22,394
BEE049	8614332	0,790	0,064	0,530	2,936	163,321	26850114	0,340	0,114	0,590	13,188	154,188	16,124
BEE152	14781975	0,726	0,060	0,609	6,077	162,831	53816848	0,209	0,127	0,658	31,612	153,303	37,689
BEE153	9557459	0,744	0,059	0,543	3,439	162,238	38876394	0,327	0,111	0,602	19,513	153,380	22,952
BEE163	11101633	0,724	0,062	0,585	4,380	162,459	48103020	0,238	0,128	0,644	27,243	153,556	31,623
BEE172	7480370	0,693	0,047	0,619	3,253	165,221	34468096	0,182	0,109	0,663	20,711	153,299	23,964
BEE174	15292573	0,708	0,069	0,562	5,832	161,538	50307717	0,205	0,121	0,619	27,876	153,383	33,708
BEE176	11677843	0,729	0,055	0,598	4,674	162,071	39096839	0,237	0,104	0,670	23,040	153,484	27,714
BEE177	10997949	0,766	0,062	0,551	3,918	161,049	34515569	0,269	0,116	0,605	18,085	153,794	22,003
BEE190	10726503	0,796	0,064	0,537	3,692	163,885	33598043	0,378	0,120	0,598	16,318	153,998	20,011
BEE191	6055388	0,833	0,064	0,521	1,948	162,587	14757467	0,377	0,107	0,594	7,179	155,071	9,127
BEE192	6528502	0,806	0,062	0,504	2,085	163,119	21001357	0,411	0,113	0,549	9,183	154,200	11,268
BEE193	6145356	0,824	0,067	0,510	1,954	162,975	17434203	0,430	0,117	0,551	7,577	154,462	9,530
BEE194	7435272	0,818	0,061	0,560	2,604	162,739	19402838	0,328	0,107	0,638	10,410	154,608	13,014
BEE195	6887512	0,811	0,059	0,542	2,355	163,167	20768851	0,359	0,099	0,616	10,555	154,517	12,910
BEE243	2408720	0,599	0,036	0,388	0,746	175,276	15452830	0,162	0,100	0,371	5,245	152,942	5,990
BEE244	4493003	0,791	0,061	0,536	1,554	164,153	16340055	0,359	0,122	0,581	7,801	154,089	9,356
BEE245	9030446	0,807	0,063	0,552	3,155	163,165	23494047	0,281	0,114	0,625	12,692	154,744	15,847
BEE246	6606412	0,843	0,068	0,527	2,116	161,448	17510158	0,348	0,118	0,594	8,684	155,479	10,800
BEE247	5349295	0,844	0,074	0,509	1,643	160,640	14397938	0,400	0,130	0,557	6,471	155,148	8,114
BEE248	4045813	0,833	0,065	0,513	1,277	162,198	10184154	0,418	0,110	0,550	4,462	154,950	5,739
BEE249	4646831	0,810	0,063	0,457	1,344	163,601	14903320	0,420	0,115	0,494	5,840	154,397	7,183
BEE250	5133027	0,812	0,063	0,500	1,612	162,613	15908544	0,444	0,109	0,541	6,711	154,227	8,323
BEE251	8840707	0,815	0,063	0,509	2,817	162,530	23795123	0,381	0,114	0,570	11,023	154,297	13,839
BEE252	8477852	0,789	0,060	0,554	3,013	163,030	27759739	0,333	0,110	0,618	14,342	154,220	17,355
BEE253	7376703	0,813	0,064	0,541	2,493	161,961	20934610	0,338	0,109	0,608	10,643	154,794	13,136

**Fig 5:**
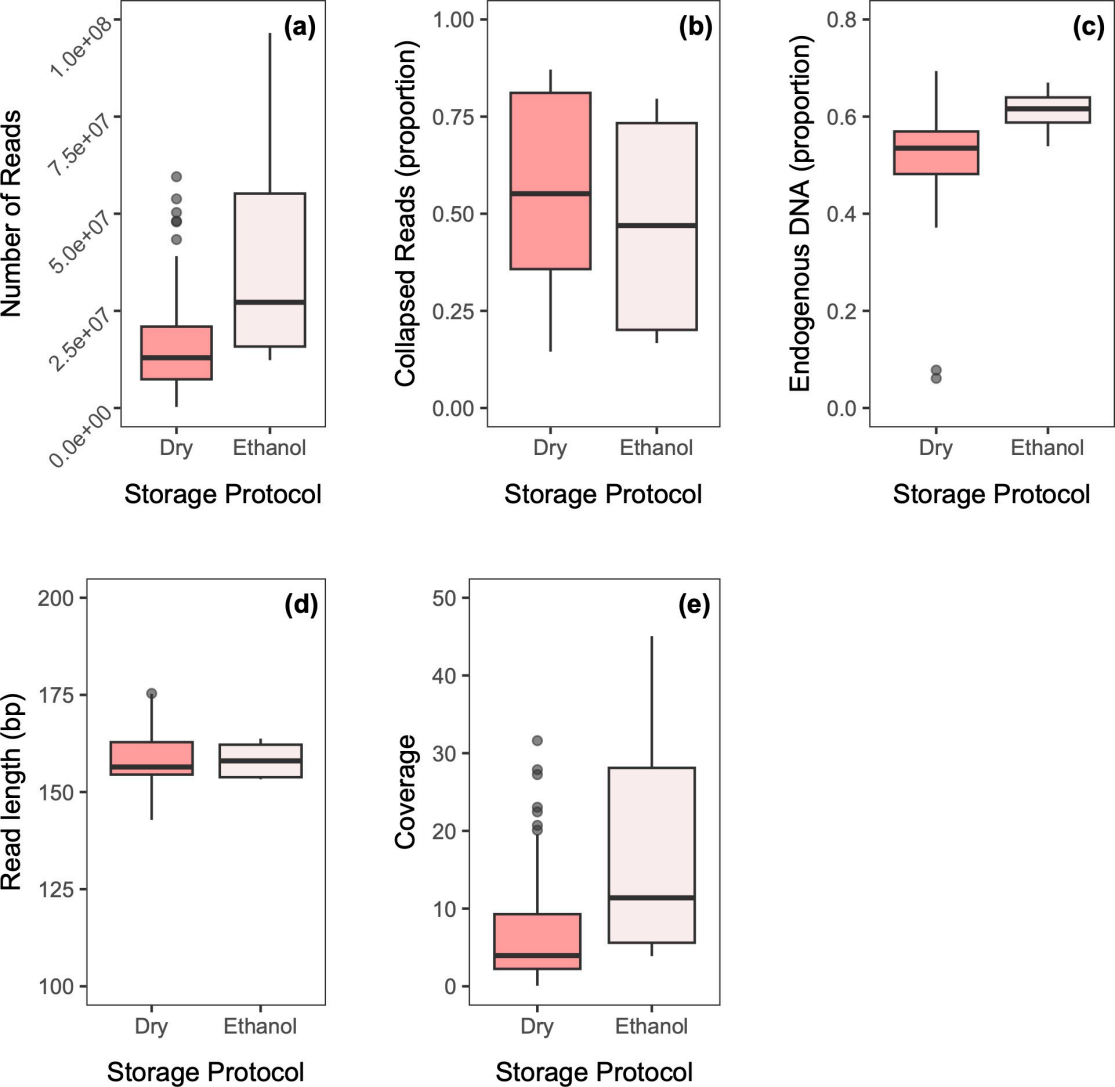
Sequencing comparisons of two combined pools of 90 bumblebee libraries from the same specimens stored using 2 different methods. Bumblebee specimens were sampled between 2017 and 2022 from various locations in Norway, Germany, Denmark and Sweden and were either stored dry at room temperature (*n* = 82) or in ethanol at -18°C (*n* = 8). The box plots compare **(a)** number of reads; **(b)** proportion of collapsed reads; **(c)** endogenous DNA proportion (unique reads only); **(d)** read length and **(e**) coverage (fold).

Ethanol-stored samples consistently produced preferable sequencing statistics, with significantly more paired reads (W = 553, P = <0.001; Fig 5A and see S1 Table), fewer collapsed reads (W = 1850, P = 0.007; Fig 5B), higher endogenous DNA proportions (W = 402, P = <0.001; Fig 5C), and similar read length (Fig 5D), leading to significantly higher overall coverage (W = 598, P = <0.001; Fig 5E).

Finally, when combining the sequencing data of Pool SP and Pool S4, we obtain at least 3-fold overall coverage for 88 out of 90 samples, and at least 10-fold overall coverage for 58. (Table 2). Sequences exhibited minimal sequencing bias as a result of Tn5 library preparation (see S1 Fig).

## Discussion

We here use an affordable and practical tagmentation-based genomic library preparation protocol to generate successful libraries from 6ng DNA extracted from entomological samples. We reduce costs by transposase enzyme dilution (similar to [25]), and pooling post-amplification libraries without quantification prior to clean-up. We also improve sequencing efficiency by applying a double-sided post-amplification clean-up protocol, selecting a narrow range of library fragments from the broad distribution of fragment sizes produced by the tagmentation reaction. We thus generated medium coverage (>3-fold) genomes for 88 out of 90 specimens, with an average of approximately 10-fold coverage. We estimate the overall economic cost for library preparation and PCR amplification of 96 reactions to be below 450 Euros. Below, we discuss a number of practical considerations.

First, initial extractions of bumblebee samples stored either dry or in ethanol revealed that dry-stored samples produced significantly higher overall DNA yields (up to 3-fold higher, see Fig 1); in fact, few extracts of ethanol-stored samples exhibited DNA concentrations beyond 1ng/μL. Assuming the dry-stored samples to be of higher quality, we chose to prioritise these samples for subsequent DNA extraction, library preparation and sequencing, resulting in the unbalanced sample sizes of the two groups (82 dry-stored samples, 8 ethanol-stored samples; see Table 1). Nonetheless, the eight extracts from ethanol-stored samples performed significantly better during sequencing, despite normalising the concentrations of all samples. Ethanol-stored samples obtained significantly more read pairs, which were also less often fully collapsed, indicating larger DNA fragments. They also yielded significantly greater proportions of endogenous DNA than the dry-stored sample sequences. We speculate that the combination of submerging samples in 96% ethanol with a storage temperature of -18°C reduces the presence of microbial or fungal contaminants, improves the proportion of endogenous DNA and decreases fragmentation of the little amount of DNA remaining. Given that our DNA library preparation protocol is of an adequate sensitivity to produce viable libraries from such low DNA amounts, we conclude that 96% ethanol-based storage of entomological specimens is preferable for high-throughput sequencing compared to dry storage, and that DNA yield is not a determining factor of library sequencing quality.

In order to increase affordability of library preparation, a 6X Tn5 transposase dilution was used, along with direct pooling of all amplified libraries prior to clean-up. As a result of reduced enzyme concentration, the tagmentation reaction produced a broad distribution of DNA library fragment sizes, with a large proportion of long fragments (see Figs 2B and 3, Pool SP). The ideal library fragment size distribution for Illumina DNA sequencing peaks tightly around approximately 600 bp [37]; in addition, long fragments have been found to produce significantly more error rates and reduced base qualities when sequenced on Illumina platforms [38]. In order to narrow library fragment size distribution around the recommended optimal length of 600 bp and thus increase sequencing efficiency, a more stringent double-sided post-amplification clean-up was performed on Pool S4, which removed both very short (<~400 bp) and very large (>~1000 bp) fragments. Consequently, Pool S4 produced significantly fewer collapsed reads, more endogenous reads and thus yielded approximately 1.5-fold more nucleotides per paired read compared to Pool SP. We therefore conclude that this stringent double-sided clean-up leads to a considerable improvement in sequencing efficiency.

To further reduce financial costs and workload, we pooled all libraries after PCR amplification and omitted individual quantification, which likely increased the variation and spread of coverage obtained by our sequences. Nonetheless, when combining the sequencing data of Pool SP and Pool S4, we find that 88 out of 90 samples have coverage of at least 3X, and 58 out of 90 samples with coverage over 10X. The vast majority of our samples are therefore suitable for a range of downstream analytical approaches, in spite of this pooling approach. While this 96-sample library preparation protocol (including PCR) runs in just a few hours, there is potential for increased efficiency through automation. The direct-post amplification pooling and lack of intermediate clean-up steps increases the ease with which this adaptation could be made.

Finally, we reiterate that 90 successful libraries were generated from a standardised DNA input of 6ng extracted from just one or two bumblebee legs. This low tissue volume minimises morphological damage to biologically valuable entomological specimens, helps to maintains their potential for future taxonomic, ecological and DNA-based research, and as a result reduces the requirement for additional entomological sampling efforts. In addition, library preparation protocols with low DNA input volumes are essential for the genomic sequencing of much smaller organisms than bumblebees, which may yield very little DNA even when entire specimens are used for DNA extraction. We therefore hope that this protocol can be of considerable value for the future of entomological genomics and monitoring.

## Supporting information

S1 Protocol Laboratory protocol describing Tn5 transposase tagmentation-based library preparation in 96-well plates, PCR and post-amplification clean-up. (DOCX)

S1 Table Sequencing statistics for two sequencing pools (SP and S4) of 90 specimens of two bumblebee species: (a) *Bombus lapidarius* (*n* = 46) and (b) *Bombus pascuorum* (*n* = 44). Bumblebee specimens were sampled between 2017 and 2022 from various locations in Norway, Germany, Denmark and Sweden, and samples were stored either dry at room temperature, or submerged in ethanol at -18°C. Libraries for both pools were generated simultaneously. The SP pool was cleaned up with a 0.6 DNA/bead ratio and was sequenced on an Illumina Novaseq SP flowcell. The S4 pool was cleaned up using a double-sided size selection protocol, with a 0.5 DNA/bead ratio clean-up followed by a 0.65 DNA/bead ratio clean-up (twice) and was sequenced on ¼ Illumina Novaseq S4 flowcell. Metadata presented for each sequencing pool are specimen ID, storage protocol, year sampled, number of reads (bp), collapsed reads (proportion), clonality (proportion), endogenous DNA (proportion), nuclear coverage (fold), mitochondrial clonality (proportion), mitochondrial coverage (fold) and read length (bp). Also presented is combined per-specimen coverage of both sequencing runs.
(XLSX)

S1 Fig Read fragmentation and nucleotide misincorporation patterns of sequencing read data from two representative libraries aligned to either the *Bombus pascuorum* (BEE008) or *Bombus lapidarius* (BEE009) assemblies. While Tn5 transposase can cause sequence bias, this bias is associated with the first 10 bases of the reads only. We find that by using simple post-analysis filtering, preliminary data (not shown) indicate that these biases do not impact our ability to perform detailed population genetic analyses. Patterns were obtained using MapDamage v. 2.0.6 after down-sampling BAM files to 1,000,000 reads (Jónsson, H., Ginolhac, A., Schubert, M., Johnson, P. L. F., & Orlando, L. MapDamage2.0: Fast approximate Bayesian estimates of ancient DNA damage parameters. Bioinformatics. 2013;29(13): 1682–1684).
(TIF)
