# Supplementary figures and images for: High-throughput sequencing of insect specimens with sub-optimal DNA preservation using a practical, plate-based Illumina-compatible Tn5 transposase library preparation method
Source: PLoS One. 2024 Mar 22;19(3):e0300865. doi: 10.1371/journal.pone.0300865 (PMC10959394; doi:10.1371/journal.pone.0300865)

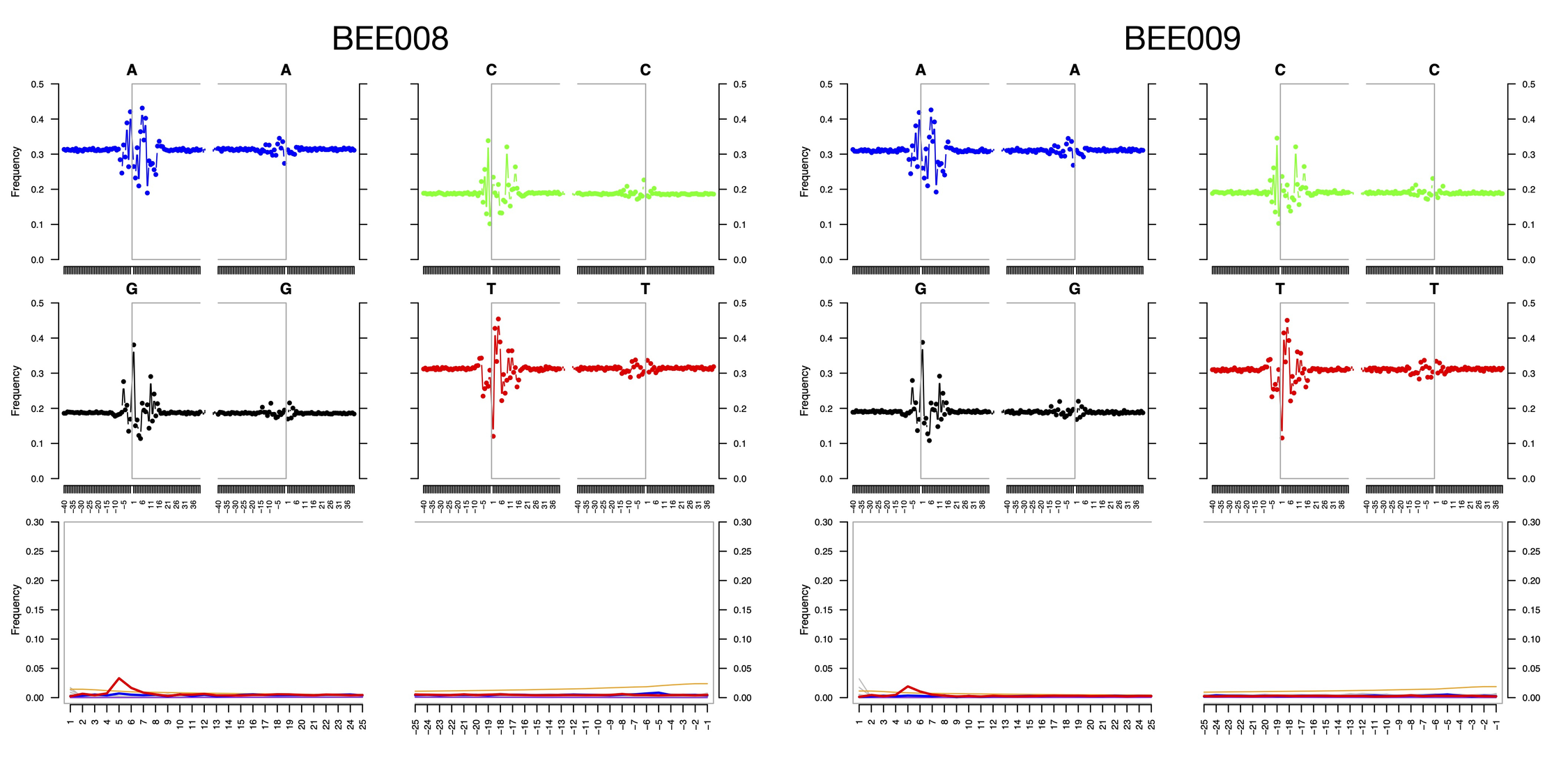

Supplement: S1 Fig — While Tn5 transposase can cause sequence bias, this bias is associated with the first 10 bases of the reads only. We find that by using simple post-analysis filtering, preliminary data (not shown) indicate that these biases do not impact our ability to perform detailed population genetic analyses. Patterns were obtained using MapDamage v. 2.0.6 after down-sampling BAM files to 1,000,000 reads (Jónsson, H., Ginolhac, A., Schubert, M., Johnson, P. L. F., & Orlando, L. MapDamage2.0: Fast approximate Bayesian estimates of ancient DNA damage parameters. Bioinformatics. 2013;29(13): 1682–1684). (TIF) [file pone.0300865.s003.tif]
